# Advanced Thymoma Successfully Treated with Cisplatin, Doxorubicin, and Methylprednisolone Therapy as Induction Chemotherapy: Two Surgical Cases and a Review of the Literature

**DOI:** 10.70352/scrj.cr.25-0439

**Published:** 2025-09-10

**Authors:** Ayuna Sato, Yukiko Matsui, Takahide Toyoda, Yuki Sata, Terunaga Inage, Kazuhisa Tanaka, Junichi Morimoto, Masako Chiyo, Hidemi Suzuki

**Affiliations:** 1 Department of General Thoracic Surgery, Chiba University Graduate School of Medicine, Chiba, Chiba, Japan; 2 School of Medicine, Chiba University, Chiba, Chiba, Japan

**Keywords:** advanced thymoma, induction chemotherapy, multimodal treatment

## Abstract

**INTRODUCTION:**

Multimodal treatment is required for advanced thymic epithelial tumors. However, to date, no pharmacotherapy has been established. Herein, we present 2 cases in which preoperative cisplatin, doxorubicin, and methylprednisolone (CAMP) therapies were effective.

**CASE PRESENTATION:**

Case 1: A woman in her 70s was diagnosed with clinical stage IVA thymoma with intrathoracic dissemination. The patient received 2 cycles of induction therapy with CAMP, resulting in a partial response. Robot-assisted thoracoscopic resection of a mediastinal tumor and pleural lesions was successfully performed. Pathology revealed a type B2 thymoma (ypT1aN0M1a, stage IVA). Case 2: A woman in her 70s was diagnosed with clinical stage IIIA thymoma invading the upper lobe of the left lung. Two cycles of CAMP therapy resulted in sufficient tumor shrinkage to allow resection of only the upper division of the left lung along with resection of the thymoma, thereby avoiding lobectomy. Pathology confirmed type B2 thymoma (ypT3N0M0, stage IIIA), and adjuvant radiotherapy was administered postoperatively.

**CONCLUSIONS:**

CAMP therapy was effective as induction chemotherapy in 2 cases of advanced thymoma, allowing for less invasive surgical approaches. Following this regimen, minimally invasive surgery was performed in one case, and the extent of pulmonary resection was reduced in the other. Therefore, this regimen may offer functional preservation and serve as a useful option for multimodal treatment.

## Abbreviations

ADOC
: doxorubicin, cisplatin, vincristine, and cyclophosphamide
CAMP
: cisplatin, doxorubicin, and methylprednisolone
FDG
: ^18^F-fluorodeoxyglucose
PAC
: cisplatin, doxorubicin, and cyclophosphamide

## INTRODUCTION

Multimodal treatment is required for advanced thymic epithelial tumors.^1,2)^ Several chemotherapeutic regimens for thymomas, including doxorubicin, cisplatin, vincristine, and cyclophosphamide (ADOC), cisplatin, doxorubicin, and cyclophosphamide (PAC), and cisplatin, doxorubicin, and methylprednisolone (CAMP), have been reported.^1–3)^ However, no effective treatment has been established. Herein, we present 2 cases in which CAMP therapy was effective. In both cases, chemotherapy was an advantage during surgery.

## CASE PRESENTATION

### Case 1

**Fig. 1:**
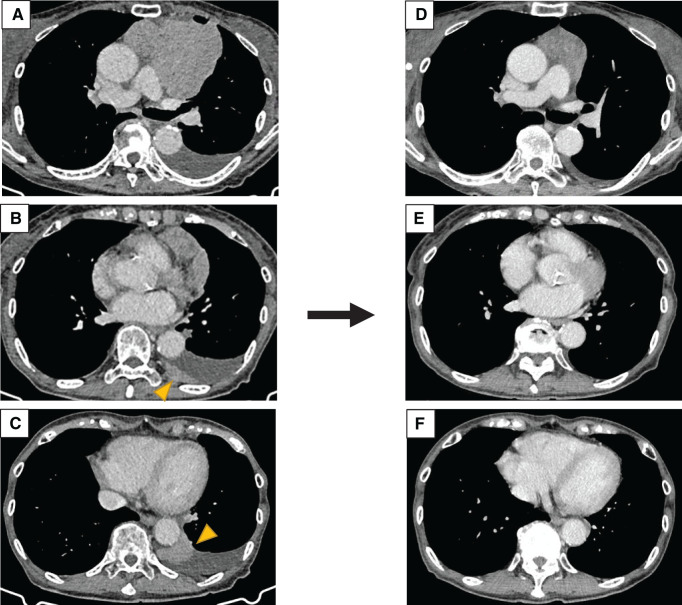
(**A**) Chest CT before chemotherapy showing a 10-cm diameter mass in the anterior mediastinum. Left pleural effusion due to dissemination was observed. (**B**) At least 3 sites of pleural dissemination were observed in the left thoracic cavity. This image shows pleural dissemination on the dorsal side of the left thoracic cavity (arrowhead). (**C**) Disseminated lesions were also observed around the descending aorta (arrowhead). (**D**) After 2 cycles of CAMP therapy, the primary tumor shrank to 5.1 cm. Pleural dissemination also shrank, and the left pleural effusion decreased. (**E**, **F**) The disseminated lesions have almost disappeared. CAMP, cisplatin, doxorubicin, and methylprednisolone

A woman in her 70s was referred to our hospital with a complaint of exertional dyspnea. Chest CT showed a 10-cm mass in the anterior mediastinum. In addition, at least 3 pleural nodules and effusion owing to dissemination were also observed in the left thoracic cavity (**Fig. 1A**–**1C**). ^18^F-fluorodeoxyglucose (FDG) PET-CT showed high accumulation of FDG in the tumors, with maximal standardized uptake values of 4.25 and 3.18. Preoperative anti-acetylcholine receptor antibody level was 14 nmol/L. A neurologist ruled out myasthenia gravis. Based on CT-guided percutaneous needle biopsy, the tumor was pathologically diagnosed as a thymoma (clinical T3N0M1a, stage IVA).

Because complete surgical resection was deemed difficult, we planned tumor reduction surgery following induction chemotherapy. The patient underwent 2 cycles of preoperative CAMP therapy, which resulted in a partial response; the primary tumor decreased in diameter to 5.1 cm, and pleural dissemination significantly regressed. The left pleural effusion and disseminated lesions also decreased (**Fig. 1D**–**1F**). The mediastinal tumor and disseminated lesions were resected using robot-assisted thoracoscopic surgery. The mediastinal pleura and pericardium were also resected. The surgery was performed in the right lateral position with 5 ports. During the surgery, we were able to observe the thoracic cavity extensively and in detail. The tumor resection was also performed appropriately. We resected 5 visible pleural disseminations, which were more than we had counted before surgery. All visible lesions were removed. Since the remaining lesions were thought to be microscopic, we performed debulking surgery.

Histopathological analysis confirmed type B2 thymoma (ypT1aN0M1a, stage IVA). Pathological findings revealed tumor invasion of the mediastinal fat, but no invasion of the mediastinal pleura or pericardium.

The patient remained alive without recurrence at 6 months postoperatively.

### Case 2

**Fig. 2:**
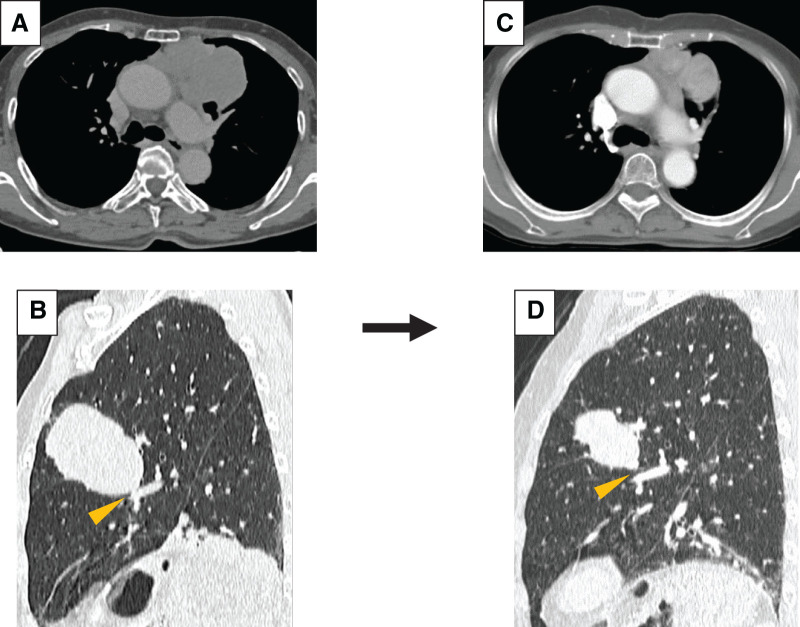
(**A**) Chest CT showing a 7-cm diameter mass in the anterior mediastinum. (**B**) Sagittal section of chest CT image. The tumor had extensive infiltration in the left upper lobe and involved A5 (arrowhead). (**C**) After 2 cycles of CAMP therapy, the primary tumor shrank to 6.0 cm. (**D**) Extent of the infiltration in the left upper lobe was reduced and A5 was free (arrowhead). CAMP, cisplatin, doxorubicin, and methylprednisolone

A woman in her 70s was referred to our hospital after detection of an abnormal shadow on chest radiography. Chest CT revealed a 7-cm diameter mass in the anterior mediastinum, which was suspected to have extensively infiltrated into the left upper lobe. The tumor had also involved A5 (**Fig. 2A** and **2B**). FDG-PET/CT showed high accumulation of FDG in the tumors, with a maximum standardized uptake value of 4.51. The preoperative anti-acetylcholine receptor antibody levels were <0.2 nmol/L. Based on a CT-guided transpercutaneous needle biopsy, the tumor was pathologically diagnosed as a thymoma with clinical T3N0M0, stage IIIA. We initially considered the need for left upper lobectomy because the tumor had extensive infiltration in the left upper lobe and involved A5. Therefore, 2 cycles of preoperative CAMP therapy were administered to reduce the size of the tumor. Following the chemotherapy, the tumor size reduced to 6.0 cm, enabling lingula preservation (**Fig. 2C** and **2D**). Mediastinal tumor resection with resection of the left upper division was performed. The surgery was performed through median sternotomy with an L-shaped incision in the left fourth intercostal space. Because of tumor shrinkage, we avoided upper lobectomy. Histopathological analysis confirmed type B2 thymoma (ypT3N0M0, stage IIIA). The tumor invaded the lungs through the mediastinum. Because the tumor was exposed in a portion of the peritumoral fatty tissue, adjuvant radiation therapy (54 Gy) was administered from 3 months after surgery. No recurrence was detected for 15 months postoperatively.

## DISCUSSION

Complete surgical tumor resection is recommended for patients with clinical stage III thymoma. In cases in which complete resection is difficult to achieve, multidisciplinary treatment is recommended.^4–6)^ However, the optimal strategy for the treatment of clinical stage IV thymoma remains controversial.^7,8)^ Debulking surgery for unresectable thymoma may be associated with improved overall survival and should be considered for patients with unresectable thymoma.^9)^

In case 1, robotic surgery was performed. There are various approaches (left side, subxiphoid, etc.) for robotic surgery. In this case, we approached from the left thoracic cavity using 5 ports in the right lateral position because the tumor and dissemination nests were relatively widespread in the left thoracic cavity. The choice of approach should depend on the location of the tumor and the extent of its invasion.

Although no consensus has been reached on chemotherapeutic regimens for thymomas owing to the lack of comparative studies, several anthracycline-based regimens, such as ADOC and CAMP regimens, have been reported.^1–3)^ Yokoi et al. reported that the response rate to CAMP therapy in patients with invasive thymomas was 92.9%, and multimodality therapy with CAMP resulted in 5- and 10-year overall survival rates of 80.7%.^10)^ In our cases, preoperative CAMP therapy resulted in tumor shrinkage in both patients.

We reviewed previous studies that evaluated the efficacy of CAMP therapy^1,4,8,10–12)^ (**Table 1**). Several reports have indicated the efficacy of CAMP therapy for the treatment of thymomas with pleural dissemination. Nakamura et al. demonstrated that the induction of CAMP chemotherapy and surgical resection in patients with thymoma having pleural dissemination resulted in a response rate of 78.9%, 5-year overall survival rate of 76.7%, and 5-year progression-free survival rate of 55.1%, indicating that the regimen was both effective and feasible.^8)^ In case 1, the pleural disseminated lesions shrank markedly after the CAMP therapy, which is consistent with previous findings. Substantial tumor size reduction has enabled robot-assisted thoracoscopic surgery. In case 2, chemotherapy reduced the extent of pulmonary resection, and we achieved complete resection macroscopically.

**Table 1:** Reports of multidisciplinary treatments with CAMP for advanced thymic epithelial tumors

First author	Number of CAMP therapy cases	Masaoka stage	Pathological subtype	5-year OS (%)
Kim ES^1)^	22	III–IVB	Epithelial, lymphocytic, mixed	95
Yokoi K^10)^	14	III–IVB	B2, B3	80.7
Ishikawa Y^11)^	7	IVA–IVB	B2, B3	81
Nakamura S ^8)^	19	IV or recurrent	B1, B2, B3	76.7
Makita A^12)^	1	IIIA	B2	NS
Our cases	2	IIIA–IVA	B2	NS

CAMP, cisplatin, doxorubicin, methylprednisolone; OS, overall survival; NS, not specified

Toxicity must also be considered when administering treatment. Neutropenia is a primary adverse event associated with CAMP therapy.^10,12)^ Clinicians should also pay close attention to potential side effects, particularly cardiac toxicity.^13)^ Mild cardiac dysfunction following postoperative radiotherapy has been reported, probably due to the synergistic effects of doxorubicin and radiation.^10)^ In the present case, during and after chemotherapy, mild edema, weight gain, and grade 3 neutropenia were observed in case 1, while grade 3 neutropenia, grade 2 edema, and grade 2 constipation were observed in case 2. All the adverse events were manageable.

In the current case, CAMP therapy was effective and feasible as induction chemotherapy. The 10-year overall survival rates of patients with clinical stage III or IVA thymoma who undergo multimodal treatment are high, allowing for the expectation of long-term survival. However, the recurrence rate is high, and long-term follow-up is essential.^10)^

The limitation of this report is that the prognosis is unclear due to the short observation period. Despite this limitation, we chose the CAMP regimen for advanced thymoma because previous reports have documented the effectiveness of this treatment, the high response rate, and its well-balanced toxicity profile compared with ADOC and PAC therapy.^10,13)^

## CONCLUSIONS

We encountered 2 cases of advanced thymoma that were successfully treated with multimodal induction CAMP therapy. Following chemotherapy, minimally invasive surgery was performed in one patient, and the extent of pulmonary resection was reduced in the other. Induction chemotherapy for advanced thymomas may be effective and has the potential for functional preservation.

## DECLARATIONS

### Availability of data and materials

The datasets supporting the conclusions of this article are included within the article and its additional files.
